# Aspergillus fumigatus during COPD exacerbation: a pair-matched retrospective study

**DOI:** 10.1186/s12890-018-0611-y

**Published:** 2018-04-03

**Authors:** Xunliang Tong, Anqi Cheng, Hongtao Xu, Jin Jin, Yimeng Yang, Sainan Zhu, Yanming Li

**Affiliations:** 1 Department of Geriatrics, Beijing Hospital, National Center of Gerontology, Beijing, 100730 People’s Republic of China; 2 Tobacco Medicine and Tobacco Cessation Centre, Center of Respiratory Medicine, China-Japan Friendship Hospital; WHO Collaborating Centre for Tobacco Cessation and Respiratory Diseases Prevention; National Clinical Research Center for Respiratory Diseases, Beijing, 100029 People’s Republic of China; 3 Department of Laboratory Medicine, Beijing Hospital, Beijing, 100730 People’s Republic of China; 4 Department of Respiratory and Critical Care Medicine, Beijing Hospital, Beijing, 100730 People’s Republic of China; 5 Statistics Department, First Hospital of Peking University, Beijing, 100034 People’s Republic of China

**Keywords:** COPD, Aspergillus fumigatus, Fungal colonization

## Abstract

**Background:**

Recently awareness of the importance of Aspergillus colonization in the airway of patients with chronic obstructive pulmonary disease (COPD) was rising. The aim of this study was to investigate the clinical features and short-term outcomes of COPD patients with Aspergillus colonization during acute exacerbation.

**Methods:**

A pair-matched retrospective study on patients presenting with COPD exacerbation was conducted from January 2014 to March 2016 in Beijing Hospital, China.

**Results:**

Twenty-three patients with Aspergillus colonization and 69 patients as controls, diagnosed of COPD exacerbation, were included in this study at a pair-matched ratio of 1:3. In stable stage, the percentage of patients with high-dose corticosteroids inhalation in the Aspergillus colonization group is higher than that of in control group (65.5% vs 33.3%, *p* = 0.048). Multivariate analysis showed that corticosteroids use was the risk factor for isolation of Aspergillus. In acute exacerbation stage, patients in Aspergillus colonization group received higher dose of inhaled corticosteroids and more types of antibiotics than control group. The short-time outcome hinted that the remission time and the duration of hospitalization were longer in the Aspergillus colonization group than in the control group (remission time: 11 ± 4 days vs 7 ± 4 days, *p* = 0.001; duration: 15 ± 5 days vs 12 ± 4 days, *p* = 0.011).

**Conclusions:**

Aspergillus colonization in the lower respiratory tract of COPD patients showed typical clinical manifestations, affected their short time outcome and provided a dilemma of clinical treatment strategy.

**Electronic supplementary material:**

The online version of this article (10.1186/s12890-018-0611-y) contains supplementary material, which is available to authorized users.

## Background

The prevalence of chronic obstructive pulmonary disease (COPD) is rapidly growing and is associated with significant morbidity and mortality, which increases economic health burden [1–3]. COPD is characterized by irreversible airflow obstruction with underlying emphysema and small airway obliteration, which commonly co-exist. Pathogenic microorganisms, such as bacteria, are commonly colonized in the airways of COPD patients, possibly contributing to increased airway inflammation, and have been implicated in COPD exacerbations [4–6]. However, fungal colonization and its potential role in acute exacerbations of COPD (AECOPD) are poorly understood.

Aspergillus spp. is a ubiquitous fungus in the environment with high sporulation capacity [7, 8]. After Aspergillus sporulates, conidia with a diameter of 2–3 μm are released into the air, enter the airway, and reach alveoli [9]. Therefore, the lung is the main organ affected by Aspergillus. Isolation of Aspergillus spp. from lower respiratory tract (LRT) samples (e.g. sputum, bronchial aspirate, or bronchoalveolar lavage) provides important etiologic evidence for its identification. Aspergillus spp. causes various diseases in lungs, such as Aspergillus colonization, Aspergillus infection and allergic bronchopulmonary aspergillus [10]. In COPD patients, impairment of the defense mechanisms of airways facilitates the binding of conidia to epithelial cells, which may cause Aspergillus colonization in the airway [11]. Positive isolation of Aspergillus spp. in LRT samples from COPD patients is common, and a previous study reported that the positive identification rate was nearly 29% [12]. However, clinical manifestations of COPD patients with Aspergillus spp. colonization from LRT have rarely been summarized and are difficult to distinguish, often leading to debate in clinical practice. Consequently, it is difficult for clinicians to make treatment strategies for Aspergillus colonization. The aims of this study were to investigate the clinical features of COPD patients with Aspergillus colonization in LRT, to analyze the risk factors that could predict the possibility of Aspergillus spp. isolation and to summarize the clinical treatment choices and outcomes of COPD patients with Aspergillus colonization.

## Methods

### Study design

This pair-matched study was conducted from January 2014 to March 2016 in the Department of Respiratory and Critical Care Medicine in Beijing Hospital, China. Patient data were collected from the electronic medical records system of our hospital and by additional chart review. The data used was part of our project “Study on Aspergillus of COPD patients”, which was approved by the ethics committee of Beijing Hospital (Approval notice number 2013BJYYEC-024-01). The informed consent was obtained from all participants in written form.

### Mentioned conditions

**Table:** 

	Conditions
A	Age between 18 and 90 years old.
B	The diagnosis of COPD was based on the Global Initiative for GOLD guidelines. COPD exacerbations are defined as an acute worsening of respiratory symptoms that result in additional therapy.
C	Positive isolation of Aspergillus spp. in an LRT sample by microbiologic examination. LRT samples included sputum, bronchial aspirate, and bronchoalveolar lavage fluid (BALF).
D	Patients without any chest CT findings of chronic pulmonary aspergillosis (CPA), invasive pulmonary aspergillosis (IPA), allergic bronchopulomnary aspergillosis (ABPA), pneumonia.

### Inclusion criteria

**Table:** 

	Definition
Aspergillus colonization	fulfilled above condition C and D above.
Aspergillus colonization group	fulfilled all conditions above, including A, B, C and D.
Control group	COPD patients who were admitted a day before or after the Aspergillus colonization group patients were recruited to the control group. At the same time, patients fulfilled above conditions A, B and D.

### Exclusion criteria

1. Immunocompromised patients, including those with allogeneic or autologous hematopoietic stem cell transplantation, neutropenia, hematologic malignant disease and solid tumor, hematologic stem cell or solid-organ transplantation, and AIDS, as were patients receiving high-dose immunosuppressive agents (e.g. for connective tissue disease and vasculitis).
2. Patients with chest CT findings of CPA, IPA, ABPA, pneumonia.
3. Patients with other underlying lung diseases.

### Microbiological examination

When patients admitted into hospital, their LRT samples were delivered for microbiological examination the next day. Microbiological examination included direct microscopy and bacterial and fungal pathogen cultvation. All samples were cultured on conventional media, including blood agar, chocolate agar, MacConkey agar and Sabouraud’s dextrose agar. Bacterial infection was defined as a colony count ≥10^5^ cfu/mL. Aspergillus and other fungal isolates were identified using microculture and standard morphological procedures.

### Data collection

Following information was collected from the electronic medical records system of our hospital: patient characteristics (sex, age, etc.), lung function, administration of corticosteroids and nutritional status before admission, clinical symptoms and signs, chest imaging and CT scan data, laboratory test results (IgE, eosinophil counts, etc.), microbiologic findings of LRT samples, treatment during hospitalization, remission time (defined as the duration of stabilization of clinical symptoms and disappearance of signs, like the typical symptoms of cough, sputum, wheeze, and the typical signs of wheezing rale), length of hospitalization or ICU stay, and mortality.

### Statistical analysis

Normally distributed continuous variables were expressed as the mean ± standard deviation and were compared with a *t*-test. Non-normally distributed continuous variables were expressed as medians and quartiles and were compared with the Mann-Whitney *U*-test. Categorical variables were compared with chi-square test or Fisher’s exact test. Logistic regression was used to identify independent risk factors for Aspergillus colonization. The first logistic regression analysis univariately considered the explanatory variables in stable stage. Only variables with *p* value < 0.10 in univariate analysis were entered in the multivariate analysis, using a backward stepwise method, with a probability value for the entry of *p* = 0.10 and removal of *p* = 0.05. Kaplan-Meier method was used to estimate the time from admission to remission of symptoms. *P* values < 0.05 were considered statistically significant. The statistical analysis was performed using SPSS 19.0.0 (IBM Corporation, Armonk, NY, USA).

## Results

### Study performance

**Fig. 1:**
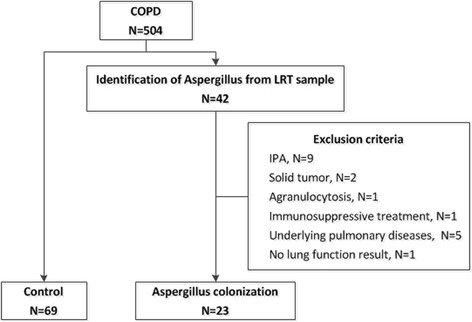
Study population selection

A total of 504 patients diagnosed with AECOPD were admitted to hospital from January 2014 to March 2016. Aspergillus spp. was identified in the LRT samples of 42 patients (all from qualified sputum specimen), resulting in a detection rate of 8.33%. According to the exclusion criteria, 19 patients with Aspergillus spp. in their sputum samples were excluded from this study, including 9 patients with IPA infection, 5 patients with other pulmonary diseases (asthma: 4 cases, bronchiectasis: 1 case), 2 patients with cancer (lung cancer: 1 case, prostate cancer: 1 case), 1 patient being treated with immunosuppressive agents for connective tissue disease, 1 patient with agranulocytosis and 1 patient who was unable to participate lung function test. Finally, 23 patients with Aspergillus colonization were included in the study group. After performing matching at a ratio of 1:3 (one study group patient to three control group patients), a total of 92 patients were enrolled in this study shown in Fig. 1.

### Demographic characteristics and treatment during stable stage

Patients in Aspergillus colonization group consisted of 20 men and 3 women with a median age of 76 ± 7 years. Most of these patients were current smokers with a smoking duration of more than 40 pack-years. All patients were classified by GOLD stage severity. The forced expiratory volume 1 (FEV_1_) of patients in Aspergillus colonization group was worse according to GOLD severity classification than that of patients in control group (FEV_1_% predicted: 40.0% ± 16.0% vs 44.5% ± 16.8%). Patients in Aspergillus colonization group also had more underlying diseases, including hypertension, diabetes mellitus and coronary heart disease, than patients in control group. The percentage of inhaled corticosteroid (ICS) use during the stable stage was higher in Aspergillus colonization group than that of control group (73.9% vs 40.6%, *p* = 0.008). Besides, more patients received a daily dose of beclomethasone greater than 1000 μg (30.4% vs 11.6%, *p* = 0.048), which was significantly different from patients in control group. The demographic characteristics of patients in Aspergillus colonization and control groups were listed in Table 1.

**Table 1:** Basic information of COPD patients in stable stage

	Aspergillus colonization, *n* = 23	Control, *n* = 69	*p* value
Demographics			
Age, yr (IQR)	76 ± 7	75 ± 8	0.741
Gender (male/female)	20/3	60/9	1.000
BMI(kg/m^2^)	22.4 ± 4.1	23.4 ± 3.7	0.266
Smoking history, n (%)	17 (73.9%)	63 (91.3%)	0.091
Pack-years (IQR)	58 ± 39	47 ± 36	0.291
COPD characteristics (post bronchodilators)			
FEV_1_% (IQR)	40.0 ± 16.0	44.5 ± 16.8	0.261
GOLD grade			
Grade I	4 (17.4%)	15 (21.7%)	
Grade II	5 (21.7%)	21 (30.4%)	
Grade III	12 (54.5%)	30 (51.7%)	
Grade IV	2 (9.1%)	3 (5.2%)	
GOLD grade ≥ 3, n (%)	14 (63.6)	33 (56.9)	0.744
Underlying conditions, n (%)			
Hypertension	15 (65.2%)	41 (59.4%)	0.806
Diabetes mellitus	7 (31.8%)	7 (15.9%)	0.201
Coronary heart disease	7 (30.4%)	16 (23.2%)	0.580
Cerebral vascular disease	4 (17.4%)	10 (14.5%)	0.774
Chronic heart failure	8 (34.8%)	12 (17.4%)	0.089
Peptic ulcer	0	6 (8.7%)	0.331
Chronic renal failure	2 (8.7%)	3 (4.4%)	0.597
Stable period treatment			
Corticosteroids use, n (%)	17 (73.9%)	28 (40.6%)	*0.008*
daily dose of inhaled corticosteroid >1000μg (Betamethasone equal dosage)	56.5%	33.3%	*0.048*
Long term oxygen therapy	3 (13.0%)	12 (17.4%)	0.753
Times of AECOPD in previous year	1.7 ± 1.3	1.3 ± 1.1	0.186

Italicized *p*-values are statistically significant, ie. *p* <0.05

Univariable baseline analysis showed that smoking history, chronic heart failure and corticosteroids use were risk factors for Aspergillus colonization in COPD patients’ LRT. After multivariable adjustment, only corticosteroids use was independently associated with Aspergillus colonization in COPD patients’ LRT (OR 4.685, 95% CI 1.529–14.355), shown in Table 2.

**Table 2:** Univariate and multivariate logistic analysis for Aspergillus colonization

Characteristics	Univariate analysis		Multivariate analysis	
	OR (95% CI)	*p* value	OR (95% CI)	*p* value
BMI	0.929 (0.817–1.057)	0.264		
Smoking history	0.270 (0.077–0.944)	*0.040*	0.275 (0.073–1.036)	0.056
Pack-years	1.008 (0.994–1.022)	0.291		
FEV_1_% pre (post bronchodilators)	0.982 (0.951–1.014)	0.259		
GOLD grade	1.968 (0.638–6.075)	0.239		
Hypertension	1.280 (0.479–3.424)	0.622		
Diabetes mellitus	1.719 (0.593–4.983)	0.319		
Coronary heart disease	1.449 (0.507–4.139)	0.488		
Cerebral vascular disease	1.242 (0.349–4.421)	0.738		
Chronic heart failure	2.533 (0.878–7.313)	0.086	3.147 (0.978–10.130)	0.055
Chronic renal failure	2.095 (0.328–13.397)	0.435		
Corticosteroids use	4.149 (1.456–11.825)	*0.008*	4.685 (1.529–14.355)	*0.007*
Long term oxygen therapy	0.713 (0.182–2.787)	0.626		
Times of AECOPD in previous year	1.299 (0.880–1.918)	0.188		

Italicized *p*-values are statistically significant, ie. *p* < 0.05

### Clinical manifestations in the exacerbation stage

The clinical characteristics and selected laboratory abnormalities of included patients were shown in Table 3. Most patients exhibited fever, cough, dyspnea and wheezing. Wheezing and wheezing rales were the most specific symptom and sign, which were significantly common in Aspergillus colonization group (wheezing: 52.2% vs 15.9%, *p* = 0.001; wheezing rales: 65.2% vs 38.2%, *p* = 0.031). Laboratory results showed that the number and percentage of eosinophils (EOS) were significantly decreased in patients in Aspergillus colonization group compared to those in control group (EOS% > 5%: 4.3% vs 23.2%, *p* = 0.044). Arterial blood gas was measured in all patients, but no significant differences were observed between two groups. Some inflammatory markers, such as C-reactive protein (CRP) and erythrocyte sedimentation rate (ESR), and total IgE levels, were not significantly different between two groups.

**Table 3:** Characteristics and examination results of COPD patients in acute exacerbation stage

	Aspergillus colonization, *n* = 23	Control, *n* = 69	*p* value
Symptom and sign			
cough	20 (87.0%)	61 (88.4%)	1.000
dyspnea	14 (60.9%)	53 (76.8%)	0.177
wheezing	12 (52.2%)	11 (15.9%)	*0.001*
fever	12 (52.2%)	28 (40.6%)	0.344
moist rale	13 (56.5%)	27 (39.1%)	0.156
wheezing rale	15 (65.2%)	26 (38.2%)	*0.031*
Blood routine			
WBC count (× 10^9^/L)	6.45 ± 2.23	7.25 ± 3.57	0.319
NEUT count (×10^9^/L)	4.73 ± 2.07	4.94 ± 3.28	0.771
NEUT% (%)	71.2 ± 13.2	65.7 ± 13.0	0.092
EOS% < 0.5%	30.4%	23.2%	0.487
EOS% > 5%	4.3%	23.2%	*0.044*
Arterial blood gas			
Pa/Fi	317 ± 42	324 ± 59	0.625
pH	7.41 ± 0.03	7.38 ± 0.72	0.063
PaCO_2_ (mmHg)	40.3 ± 6.3	44.5 ± 10.6	0.088
PaO_2_ (mmHg)	70.5 ± 10.9	76.8 ± 15.5	0.102
Biomarker			
CRP (mg/dl)	3.23 ± 3.51	2.53 ± 3.12	0.412
ESR (mm/h)	25.3 ± 28.2	20.0 ± 20.3	0.436
Total IgE (IU/ml)	441 ± 1075	282 ± 362	0.386

Italicized *p*-values are statistically significant, ie. *p* < 0.05

### Detection of pathogenic bacteria and other fungi

To investigate whether Aspergillus colonization was associated with combined identification of other specific microbial pathogens, we compared the cultivation results of LRT samples from the Aspergillus colonization group and control group. Particular attention was paid to pathogenic bacteria identification, including Acinetobacter baumannii, methicillin-resistant *Staphylococcus aureus* (MRSA), Pseudomonas aeruginosa, Klebsiella pneumonia, Stenotrophomonas maltophilia and Enterococcus faecium. The percentage of pathogens identification in two groups showed no statistical difference (Additional file 1: Table S1). Besides, some of other fungi were also detected in our microbiologic cultivation, especially Saccharomycetes.

### Patient treatment during hospitalization and short-term outcomes

**Fig. 2:**
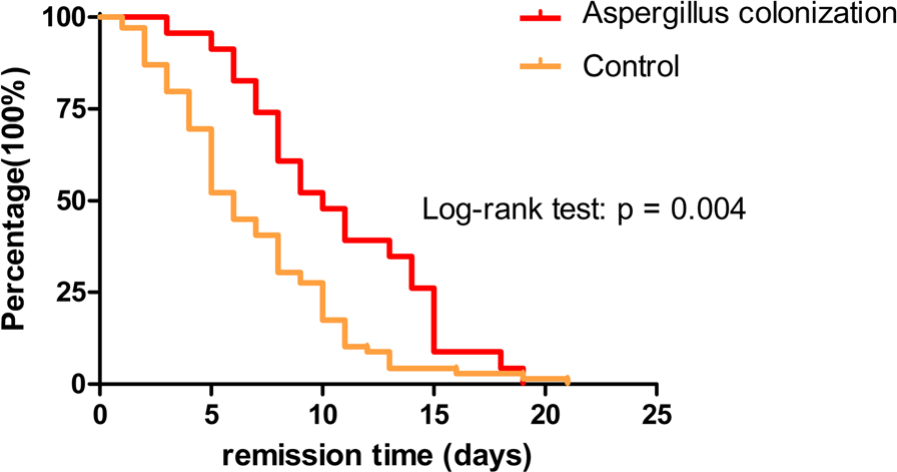
Remission time curve estimated by Kaplan-Meier analysis

The Aspergillus colonization group included a higher percentage of patients who received more than one class of antibiotics (52.2% vs 20.3%, *p* = 0.006) and had a longer duration of antibiotic usage (13 ± 5 vs 10 ± 4 days, *p* = 0.003) than that of the control group. In addition to antibiotic treatment, systemic corticosteroids and/or ICS were used to control wheezing symptom. The percentage of patients in Aspergillus colonization group who use high dose of inhaled corticosteroids (accumulated budesonide dose: no less than 40 mg) were higher than that of control group (30.4% vs 5.8%, *p* = 0.002). Remission time curve was estimated by Kaplan-Meier analysis. Median remission time was 10 days in the Aspergillus colonization group and 6 days in the control group (log-rank test *p* = 0.004) (Fig. 2). The duration of hospitalization were longer in the Aspergillus colonization group than it is in the control group (15 ± 5 days vs 12 ± 4 days, *p* = 0.011), shown in Table 4.

**Table 4:** Clinical treatment and short-term outcomes in AECOPD patients

	Aspergillus colonization, *n* = 23	Control, *n* = 69	*p* value
Admitted in ICU	4 (17.4%)	11 (15.9%)	1.000
Machined ventilation	3 (13.0%)	9 (13.0%)	0.906
Antibiotics	21 (95.5%)	66 (95.7%)	1.000
More than 1 class	52.2%	21.7%	*0.006*
Duration of treatment (days)	13 ± 5	10 ± 4	*0.003*
Corticosteroid use	17 (77.3%)	42 (60.9%)	0.204
Systemic only	0	2 (2.90%)	1.000
Inhalation only	8 (34.8%)	20 (29.0%)	0.601
Dose of usage			
Budesonide (inhaled,≥40 mg)	30.4%	5.8%	*0.002*
Methylprednisolone (iv,≥120 mg)	39.1%	31.9%	0.524
Bronchodilators (aerosol inhalation)	21 (91.3%)	57 (82.6%)	0.505
Ipratropium Bromide (mg)	16.3 ± 11.6	14.8 ± 8.2	0.631
Salbutamol (mg)	115 ± 98	83 ± 53	0.308
Remission time (days)	11 ± 4	7 ± 4	*0.001*
Duration of hospitalization (days)	15 ± 5	12 ± 4	*0.011*

Italicized *p*-values are statistically significant, ie. *p* < 0.05

## Discussion

AECOPD is often associated with infectious agents, including bacteria, virus and fungi. A previous study was conducted in critically ill patients with isolation of Aspergillus spp. from the respiratory tract, with mortality rates of 50% in the colonization group and 80% in the invasive infection group after 9 months of follow-up [13]. Therefore, clinicians usually focus on infection when a positive Aspergillus spp. culture is obtained from the LRT. However, the significance of a more frequent clinical phenomenon, Aspergillus spp. colonization, has yet to be clarified. In this research, a pair-matched observational study was conducted to investigate the differences in the clinical manifestations and short-term outcomes between COPD patients with and without Aspergillus colonization in LRT.

Cigarette smoking, one of the major risk factors for the development of COPD, induces structural and functional changes in airway epithelium in vitro and in vivo [14–16]. In our study, the number of patients with a history of smoking was higher in the Aspergillus colonization group than in the control group, which indicated that smoking is a potential risk for Aspergillus colonization. Cigarette smoking and repeated airway inflammation could alter the structure and function of lung and injure a profound effect on the host defense against invading pathogens and particulates, thus impairing the airway epithelium [17, 18] and mad COPD patients more susceptible to Aspergillus colonization. Meanwhile, most patients in the Aspergillus colonization group received higher doses of ICS during stable stage treatment, in contrast to the control group. Our findings were consistent with a previous study that suggested that high-dose corticosteroids use was a risk for Aspergillus colonization or positive Aspergillus culture [19, 20].

In our study, Aspergillus-colonized patients presented with wheezing and wheezing rales in the acute exacerbation period. More than half of the patients received systemic corticosteroids and/or ICS. The percentage of corticosteroid usage in the two groups was similar, but in the Aspergillus colonization group, patients received higher doses of ICS. This finding suggested that Aspergillus colonization contributed to an increased severity of exacerbations in COPD patients. These phenomena suggested that high-dose ICS treatment was related to Aspergillus colonization and induced similar clinical manifestations to allergic reactions due to Aspergillus colonization. A previous study on the mechanism involved in Aspergillus-related allergic reactions was based on the Aspergillus hyphae and involved antigen-triggered mast cell degranulation and release of histamine and inflammatory factors [21–24]. These data showed that Aspergillus colonization may aggravate airway hyper-responsiveness and worsen airway inflammation and bronchoconstriction. But no cohort study of patients with repeated cultures of Aspergillus have been done in COPD patients, it is unclear whether fungal colonization contributes to lower lung function or is a marker of more severe lung disease and aggressive therapy. In our study, patients with Aspergillus colonization had a longer time to be stable and a longer duration of hospitalization, which indicated that Aspergillus colonization was related with clinical manifestations and short-term outcomes of COPD patients.

After demonstrating the significance of Aspergillus colonization in the airways of COPD patients, we highlighted an essential clinical treatment dilemma: whether to eliminate colonization with anti-fungal therapy or to stabilize wheezing with continuous ICS. Because Aspergillus colonization is clinically significant, the treatment strategy should aim to eliminate colonization. However, a previous study showed that the removal of Aspergillus colonization did not improve lung function in a long-term observation; this finding cast doubt on the value of anti-fungal therapy. Due to the lack of research on the effect of Aspergillus colonization on AECOPD and stable COPD, challenges remain for clinical decision making. The relationship between colonization and invasive infection is unclear. Whether increasing the fungal load of colonization in the airway could result in invasive pulmonary mycosis has not been determined. Aspergillus colonization induces sustainable inflammation in the airway, which leads to worsening lung function. Meanwhile, poorer lung function is significantly associated with Aspergillus colonization. However, in clinical practice, ICS are commonly used to control wheezing and airway inflammation, which could also enhance the risk of Aspergillus colonization in the airway. It is concerning that once Aspergillus colonization in the airways of COPD patients is identified, a vicious cycle is established. Unfortunately, no accurate timing, biomarker, or scoring system exists that could determine the optimal antifungal therapy.

There were some limitations to this study: (1) we could not determine the timing of Aspergillus colonization: the acute exacerbation or stable stage; (2) we only conducted this retrospective study without a long-term observation from the beginning of Aspergillus colonization to its causing symptomatic clinical manifestation. Thus, whether Aspergillus colonization could affect the clinical process was still unknown, including lung function decline, the frequency of acute exacerbation, and daily symptoms; and (3) we performed this research in a single center and recruited a small sample of patients.

## Conclusions

Our findings show that Aspergillus colonization in the airway could cause significant change in clinical manifestations and treatment outcome of COPD patients. This study provides new insight for clinicians when managing patients with fungal colonization and also indicates the directions for future comprehensive studies, including the development of a reliable and rapid method to accurately identify infection and colonization and to develop a full-scale investigation and treatment strategy of COPD patients with Aspergillus colonization.

## Additional file

Additional file 1: **Table S1.** Detection of pathogenic bacteria and other fungi. (DOCX 14 kb)

## Abbreviations

ABPA: Allergic bronchopulomnary aspergillosis
AECOPD: Acute exacerbation of chronic obstructive pulmonary disease
BALF: Bronchoalveolar lavage fluid
COPD: Chronic obstructive pulmonary disease
CPA: Chronic pulmonary aspergillosis
CRP: C-reactive protein
CT: Computed tomography
EOS: Eosinophils
ESR: Erythrocyte sedimentation rate
FEV_1_: Forced expiratory volume 1
GOLD: Global initiative for chronic obstructive lung disease
ICS: Inhaled corticosteroids
ICU: Intensive care unit
IPA: Invasive pulmonary aspergillosis
LRT: Lower respiratory tract
