# Supplementary material for: Aspergillus fumigatus during COPD exacerbation: a pair-matched retrospective study
Source: BMC Pulm Med. 2018 Apr 3;18:55. doi: 10.1186/s12890-018-0611-y (PMC5883425; doi:10.1186/s12890-018-0611-y)
Supplement: Supplementary file 1 — Table S1. Detection of pathogenic bacteria and other fungi. (DOCX 14 kb) [file 12890_2018_611_MOESM1_ESM.docx]

Table S1 Detection of pathogenic bacteria and other fungi

|  | Aspergillus colonization, n=23 | Control, n=69 | *p* value |
| --- | --- | --- | --- |
| Pathogenic bacteria, n (%) | 6(26.1%) | 8(11.6%) | 0.106 |
| Acinetobacter baumannii | 0 | 1(1.4%) | 1.000 |
| Methicillin resistant staphylococcus aureus | 1(4.3%) | 2(2.9%) | 1.000 |
| Pseudomonas aeruginosa | 2(8.7%) | 1(1.4%) | 0.153 |
| Klebsiella pneumoniae | 0 | 1(1.4%) | 1.000 |
| Stenotrophomonas maltophilia bacteria | 1(4.3%) | 0 | 0.250 |
| Enterococcus | 2(8.7%) | 3(4.3%) | 0.596 |
| Other fungi, n (%) | 6(26.1%) | 17(24.6%) | 1.000 |
